# Patient-Specific Cerebral Blood Flow Simulation Based on Commonly Available Clinical Datasets

**DOI:** 10.3389/fbioe.2022.835347

**Published:** 2022-03-04

**Authors:** Yuanyuan Shen, Yanji Wei, Reinoud P. H. Bokkers, Maarten Uyttenboogaart, J. Marc C. Van Dijk

**Affiliations:** ^1^ Department of Neurosurgery, University Medical Center Groningen, University of Groningen, Groningen, Netherlands; ^2^ Faculty of Science and Engineering, University of Groningen, Groningen, Netherlands; ^3^ Department of Radiology, Medical Imaging Center, University Medical Center Groningen, University of Groningen, Groningen, Netherlands; ^4^ Department of Neurology, University Medical Center Groningen, University of Groningen, Groningen, Netherlands

**Keywords:** circle of Willis, hemodynamic model, cerebral blood flow, patient-specific simulation, cerebrovascular disease

## Abstract

Cerebral hemodynamics play an important role in the development of cerebrovascular diseases. In this work, we propose a numerical framework for modeling patient-specific cerebral blood flow, using commonly available clinical datasets. Our hemodynamic model was developed using Simscape Fluids library in Simulink, based on a block diagram language. Medical imaging data obtained from computerized tomography angiography (CTA) in 59 patients with aneurysmal subarachnoid hemorrhage was used to extract arterial geometry parameters. Flow information obtained from transcranial Doppler (TCD) measurement was employed to calibrate input parameters of the hemodynamic model. The results show that the proposed numerical model can reproduce blood flow in the circle of Willis (CoW) per patient per measurement set. The resistance at the distal end of each terminal branch was the predominant parameter for the flow distribution in the CoW. The proposed model may be a promising tool for assessing cerebral hemodynamics in patients with cerebrovascular disease.

## 1 Introduction

Fifteen percent of the total cardiac output is distributed to the brain (Burton, 1965). The circle of Willis (CoW) is a circulatory anastomosis at the base of the brain and serves as the central hub that distributes blood flow to the brain. The morphology (Kayembe et al., 1984; Chuang et al., 2008; Krasny et al., 2014) and local hemodynamic flow patterns (Li et al., 2009; Can and Du, 2015; Tuenter et al., 2016) within the CoW are associated with cerebrovascular disease, such as carotid artery stenosis and intracranial aneurysms. Numerical modeling of the blood flow through the CoW can help understand the relationship between hemodynamics in the CoW and cerebrovascular disease. However, due to the complexity of the CoW-structure and the large anatomical variation of the CoW in the population, developing a reliable patients-specific numerical model for assessing cerebral hemodynamics is a great challenge.

Various numerical models have been proposed for investigating the hemodynamics in the CoW. The zero-dimensional (0D) model is the simplest model that can provide great insight into the compensation mechanisms of the CoW (Hillen et al., 1988). One-dimensional (1D) modeling has been widely employed for studying hemodynamic effects in the CoWdue to anatomical variations and occlusions (Alastruey et al., 2007; Huang et al., 2018). The more complex three-dimensional (3D) model became popular in recent decades, thanks to the fast development of computational fluid dynamics (CFD) (Alnæs et al., 2007). Detailed flow patterns in the intracranial aneurysms can be obtained with CFD models, which could be used to investigate the rupture risk of IAs (Asgharzadeh et al., 2020). Although the numerical models are based on the rigorous derivation of the fundamental governing equations of fluid dynamics, it was highly sensitive to the model input, particularly the inlet/outlet conditions (Venugopal et al., 2007; Balossino et al., 2009; Boccadifuoco et al., 2018).

In a recent review by Berg et al. (2019) about the hemodynamics modeling in intracranial aneurysms, it was concluded that the accuracy of the pre-and post-processing (e.g., geometry reconstruction, boundary condition setting, flow field analysis) were crucial in numerical simulation. Although many high-end numerical models have been proposed for cerebral hemodynamic studies with prospective potential for clinical practice, to the best of our knowledge, no existing numerical models have been universally accepted by clinicians to directly serve as clinical tool, due to the great uncertainty in patient-specific simulation. For patient-specific modeling, the required input not only includes patient-specific geometry, but more importantly appropriate physiological parameters, as well as proper boundary conditions per patient.

Many attempts have been made to develop patient-specific cerebral blood flow models. Zhang et al. (2016) proposed a 0D-1D coupling model and iteratively adjusted the peripheral cerebral resistance to match the measurement. They demonstrated that combining multiple sources of measurement by individual patients within the hemodynamic model can provide more comprehensive flow information. Lal et al. (2017) presented a more advanced approach for non-invasive estimation of the blood flow in the cerebral arteries, using an ensemble Kalman filter (EnKF) as an optimization tool. They utilized a 3-point clinical measurement of the transient blood flow to tune 21 input parameters. As such, they provided a feasible approach to assess blood flow at non-accessible locations in the cerebral arterial tree. Helthuis et al. (2020) introduced a patient-specific cerebral blood flow model with a structured tree and a simple auto-regulatory model at the distal boundary conditions. They validated their model with 20 healthy subjects and achieved a good agreement. Although these models can provide valuable hemodynamic information for patients, their clinical applicability was limited. The difficulties are two-fold: 1) This interdisciplinary research needs close collaboration between clinicians, biomedical engineers and computer science engineers. The clinicians commonly lack awareness on the role of hemodynamics but prefer traditional medical indices for clinical decision making (Singh et al., 2009). 2) A patient-specific model requires input that may be difficult to be obtained in clinical practice. Thus, many assumptions have to be made, which reduces the reliability of the hemodynamic assessment.

The objective of this study is to investigate cerebral hemodynamics in patients with cerebral vasospasm following aneurysmal subarachnoid hemorrhage (aSAH). A numerical model of patient-specific cerebral flow simulation is proposed, using clinical data obtained from a previously published study that investigating cerebral vasospasm in subarachnoid hemorrhage (TACTICS) study van der Harst et al. (2019). Our hemodynamic model is based on a lumped model in block diagram, which can be intuitively and easily used by clinicians. TCD measurements and common medical records are integrated to correct the input parameters of the model with a three-steps calibration procedure. The proposed model is applied to reproduce 66 cases of patient-specific hemodynamics at the time that vasospasm usually may occur. The validity of the proposed model is demonstrated by comparing the results with and without calibration procedures. The importance of the parameter calibration for the patient-specific simulation is discussed.

## 2 Methodology

**FIGURE 1:**
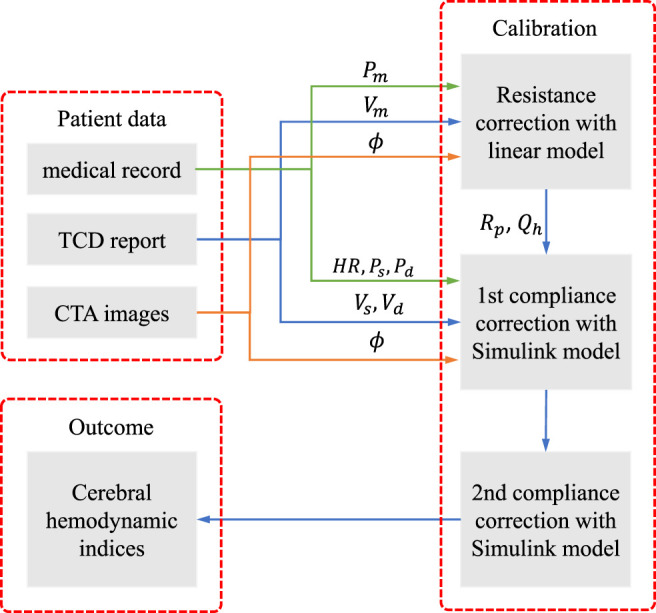
Flowchart of the numerical modelling framework.

The patients’ specific data were collected from the medical record of the prospective TACTICS study, including basic medical file, CTA images and TCD reports. The CTA and TCD were performed within 24 h of each other. The brachial mean, systolic and diastolic blood pressure (*P*_*m*_, *P*_*s*_ and *P*_*d*_), and the heart rate *HR* were obtained from the basic medical file. The geometry of the arterial network of the CoW, e.g., the diameter of arterial segments *ϕ*, was extracted from CTA images. The measured mean, systolic, and diastolic blood flow velocity (*V*_*m*_, *V*_*s*_ and *V*_*d*_) at specific segments were obtained from TCD reports. Figure 1 illustrates the steps to integrate the collected clinical data to assess patient-specific cerebral hemodynamics.

The workflow consists of three steps:

(1) determine the peripheral resistance *R*_*p*_ at each terminal branch and the periodical blood flow rate at the heart *Q*_*h*_ with a linear steady model of cerebral arterial network;
(2) correct the peripheral compliance of the body artery network and implement the tentative simulation with the Simulink model;
(3) correct the compliance of the cerebral arterial segments by adjusting the Young’s modulus of the cerebral segments and implement the final simulation with the Simulink model.

The first step is to obtain the optimal blood flow distribution at the terminal branches of the cerebral arterial network, which can match the measured blood velocity by TCD. The second step provides the background pulse pressure at the entrance of the cerebral network that is in concordance with the medical record. The third step corrects the pulse waveform from the previous step to match the systolic and diastolic blood velocity by TCD measurement. The three-steps calibration procedure aims to tune the personalized model parameters to improve the fit of model predictions against measured data. The calibration is significantly important, in order to perform patient-specific simulation to reproduce the cerebral hemodynamics at the time of measurement, as will be shown in the result section.

### 2.1 Physiological Data

From 59 adult patients, diagnosed with aSAH within 4 days after onset, 66 sets of data were obtained. The CTA and TCD measurements have been further detailed in Shen et al. (2020).

In each CTA, the diameters of 17 cerebral segments were measured by semi-automated carotid lumen segmentation. The diameters of begin and end points of each segment were measured, while the simulation was based on an uniform diameter per segment which was calculated based on equivalent circular truncated cone volume. The thickness of arterial wall was assumed to be 25*%* of its radius. This was not critical, as wall thickness in conjunction with wall elasticity determine the compliance of each segment, while wall elasticity per patient per segment in the model calibration procedure will be adapted.

In each TCD, flow velocities at 11 locations on 9 cerebral segments were measured. Although patients with aSAH are prone to experience cerebral vasospasm, the measured velocity by TCD was thought to reflect the hemodynamics in the CoW at the time of CTA, since the delay between the examinations was short. Thus, patient-specific cerebral hemodynamics under vasospasm condition could be assessed by integrating the TCD and CTA measurements in the hemodynamic model.

For other required but unknown/unavailable physiological properties, such as elastic modulus of the segments, peripheral resistance, and compliance of the terminal branches, we used the same values as that in Alastruey et al. (2007), but would make corrections for each case as described in the following sections.

### 2.2 Simulink Model

**FIGURE 2:**
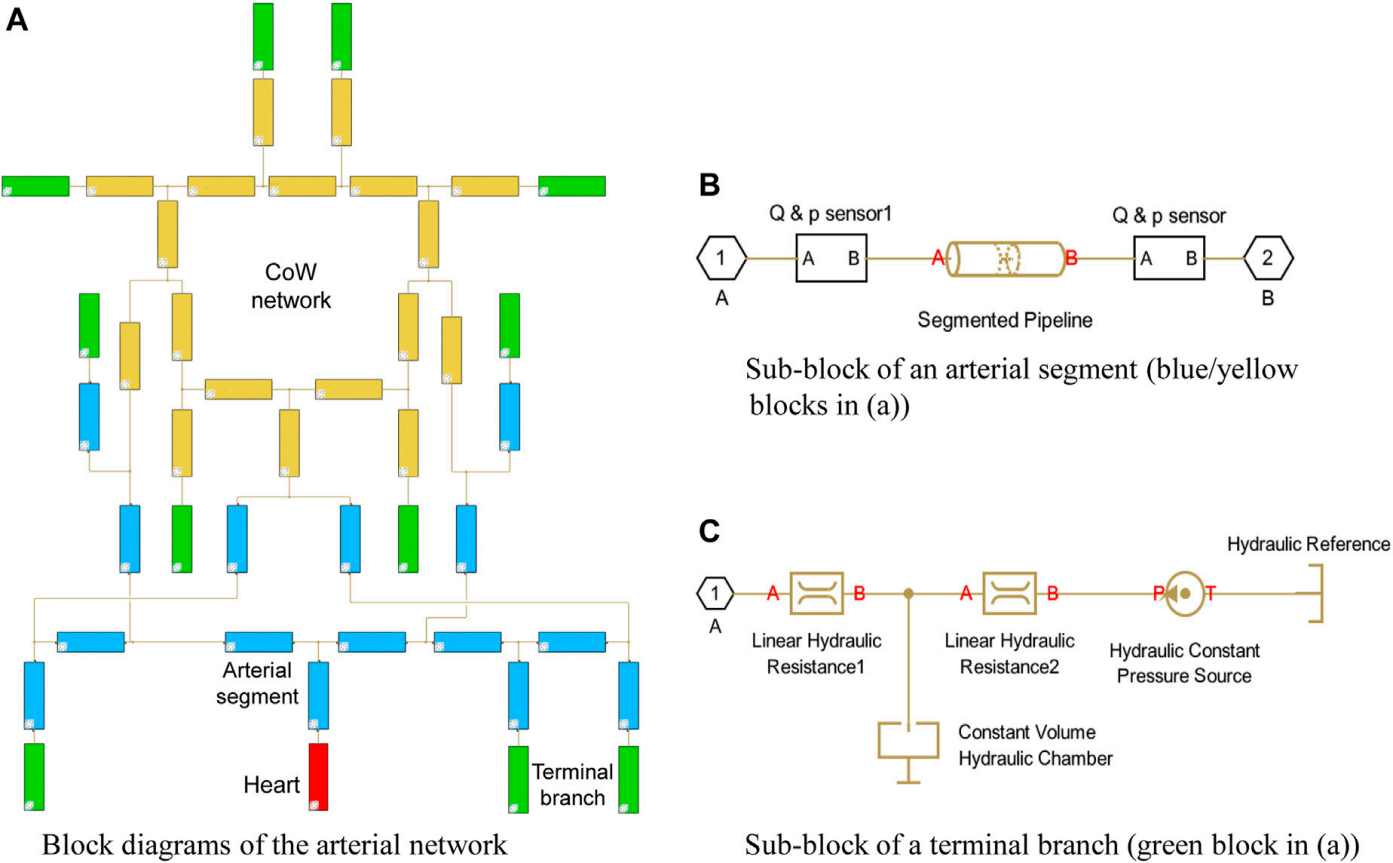
Simulink model of arterial network with 33 segments.

Our hemodynamic model was developed based on Simulink, using Simscape Fluids library, which can provide an intuitive way to model the blood circulation system as a hydraulic system. The model is based on the block diagram environment that allows to easily customize the connectivity of the arterial network, as shown in Figure 2A.In this model, each block consists of a piece of governing equations that represents a simplified hydraulic component. The blocks are linked with connectors that form a complex hydraulic system. The system is interpreted as a set of differential or algebraic equations that are solved through symbolic formula manipulation.

In the present model, the arterial system originates from the heart (the red block) and ends at the green blocks, which depict the terminal branches. Each segment is represented with an individual blue block with the numbering and name referring to Table 1 in Alastruey et al. (2007), while the segments in the CoW are highlighted in yellow. The polylines represents the interconnection between the segments, which passes the pressure and flow rate data in the network.

**TABLE 1:** ICC of various celebrate segments under various simulations.

Segment	Un-calibrated			1*st* calibrated			2*nd* calibrated		
	*Q*_*d*_	*Q*_*m*_	*Q*_*s*_	*Q*_*d*_	*Q*_*m*_	*Q*_*s*_	*Q*_*d*_	*Q*_*m*_	*Q*_*s*_
ICA	0.07	0.12	0.11	0.77	0.92	0.65	0.84	0.92	0.80
MCA	0.06	0.13	0.15	0.88	0.92	0.73	0.85	0.92	0.82
ACA	0.03	0.07	0.08	0.81	0.88	0.74	0.81	0.88	0.76
PCA	0.11	0.22	0.24	0.93	0.99	0.88	0.91	0.99	0.88
BA	0.12	0.21	0.24	0.76	0.75	0.58	0.65	0.75	0.70

A simple heart model directly enforces a periodical inlet flow rate using the “Hydraulic Flow Rate Source” block, which describes a cardiac cycle with a half-sinusoidal flow rate wave (systole) and zero in the rest of the period (diastole) that is expressed as:

$$Q_{h}\left(t\right)=\left\{\begin{array}{cc} \;\frac{\pi }{2\alpha_{T}}Q_{h,m}\sin\left(\frac{\pi t}{\alpha_{T}T_{h}}\right),\; & \text{if}\;t<t_{s}\\ 0,\; & \text{otherwise.} \end{array}\right.$$ (1)

where *α*_*T*_ is the ratio of systole duration over the duration of heartbeat, *α*_*T*_ = 0.3 is chosen in the study; *T*_*h*_ is the duration of heartbeat that is obtained from the medical report; *Q*_*h*,*m*_ is the volumetric flux of the heart, as estimated in Section 2.3.

The arterial segments are described with “Segmented Pipeline” block, which accounts its resistance, fluid inertia, and wall compliance, as shown in Figure 2B. In this block, the deformation of the segmental wall is quantified with a static pressure-diameter coefficient *K*_*p*_, which establishes the relationship between pressure and internal diameter of the arterial segment at steady-state condition. *K*_*p*_ is determined with the common properties of each arterial segment as:

$$K_{p}=\frac{\phi^{2}}{2Eh}\left(1-\sigma^{2}\right),$$ (2)

where *ϕ* is the internal diameter of the artery at the reference pressure, *E* is the Young’s modulus of the artery, *h* is the thickness of the artery; *σ* is the Poisson’s ratio, *σ* = 0.5. The pressure loss of the segment due to the friction is computed with the flow regime-dependable friction factor, which can take into account the possible flow separation due to the cerebral vasospasm. The friction factor in turbulent regime is determined with the Haaland approximation. In addition, the sensor blocks are used to monitor the pressure and flow rate at both ends of each segment.

A three-element Windkessel model (WK3) is embedded in the terminal branch to minimize the artificial reflection, see Figure 2C. A WK3 consists of two two resistances and a compliance. The “Linear Hydraulic Resistance” block is used to represent the resistance and The “Constant Volume Hydraulic Chamber” block is used to represent the peripheral compliance. Note that describing the peripheral compliance *C* in “Constant Volume Hydraulic Chamber” block is not straightforward, but using an equilibrium chamber length, which is proportional to the peripheral compliance, described as:

$$l_{eq}=\frac{2C}{\pi \phi K_{p}}.$$ (3)

The peripheral compliance and two resistances in WK3 are customized per terminal branch per patient.

From the description above, we can distinguish the present model from other lumped models. The present model directly uses the hydraulic description (pipeline network) instead of the common hydraulic-electrical analogue (electrical network). It is an object-oriented model, so the interpretation of the model layout is straightforward and intuitive. We can easily build patient-specific artery network by modifying the geometrical parameters and the connectivity of the network that makes this model suitable for investigating the cerebral hemodynamics with considerable CoW variation.

The Simulink model uses an arterial network with 33 segments (Alastruey et al., 2007) as the basis. For each patient-specific case, the diameters of the cerebral segments are altered by the measurement from the CTA images. The resistance and the compliance in each WK3 and the wall compliance of each segment are determined by calibration procedure using TCD measurement, as described in Section 2.3, 2.4.

### 2.3 Peripheral Resistance Correction

**FIGURE 3:**
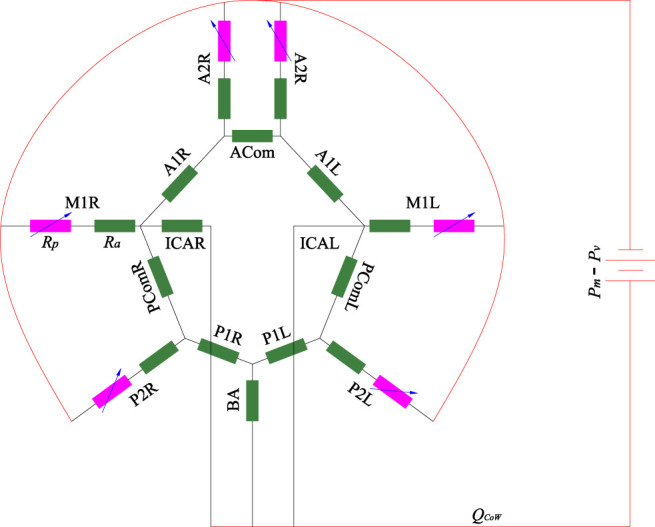
Linear steady model of CoW arterial network.

Analytical model by Hillen et al. (1988) has shown that the peripheral resistances dominate the flux distribution in the efferent segments and strongly influence the flux distribution in the afferent vessels. Hence, the peripheral resistance should be adapted to reflect the real flow situation at the time of the measurement for the patient-specific simulation. In the present study, a simple linear steady model was used to estimate the peripheral resistance, as shown in Figure 3.

The model only consisted of the CoW-segments, including three afferent arteries (left-right ICA and BA), six efferent arteries (M1L, M1R, A2L, A2R, P2L and P2R) and their interconnection. Several assumptions were made in this model: 1) The pressure drop between inlet and outlet are assumed to be equal as the difference between the mean blood pressure and the pressure at the entrance of the venous system (Δ*P* = *P*_*m*_ − *P*_*v*_, with *P*_*v*_ = 5 mmHg); 2) the flow is fully developed (steady and laminar), the resistance of the arterial segments *R*_*a*_ follows the Hagen–Poiseuille law, *R*_*a*_ = 128*η*/*πϕ* ^4^ with *η* blood viscosity; 3) the peripheral resistance *R*_*p*_ is considered a variable resistance, which may be adapted to alter the blood flow distribution in the CoW. Mathematically, the model is equivalent to an electric circuit with constant resistance and voltage source, which can be solved with node voltage analysis. Given specific peripheral resistance at the efferent arteries, the flow rate in each segment can be numerically obtained. TCD generally measures blood flow velocity of the target segments, while the exact location is unknown. Because the diameter varies along the vessel, the flow rate of the target segment cannot be directly determined. We measured the diameters at the start point *ϕ*_*s*_ and end point *ϕ*_*e*_ of the segment on CTA, thus the flow rate of the segment *Q*_*m*_ should fall within the interval $\left[0.25\pi \phi_{e}^{2}V_{m},0.25\pi \phi_{s}^{2}V_{m}\right]$ , where *V*_*m*_ is the measured mean flow velocity. The peripheral resistance can be obtained by searching for the minimum of the norm of the relative error of the flow rates,

$$f\left(R_{p}\right)=min\sqrt{\overset{N}{\underset{i=1}{\sum }}{\left(\frac{Q_{n}^{i}\left(R_{p}\right)-Q_{m}^{i}}{Q_{m}^{i}}\right)}^{2}},$$ (4)

where ***R***_*p*_ is a vector of the peripheral resistance of the six outlet arteries, $R_{p}\in R^{6}$ ; $Q_{n}^{i}$ is the flow rate obtained by the linear model; *N* is the number of TCD measurement, *N* = 9. Due to technical limitations of TCD, e.g., inadequate acoustical temporal bone window, it is possible that there is missing data on part of the locations for some cases. In these cases, *N* equals to the number of locations with available data. Eq. 4 essentially describes an optimization problem about the cerebral blood flow distribution, based on the measured velocity.

Summing up the flow rate of the three afferent arteries, we can obtain the overall blood flow rate of the CoW *Q*_*CoW*_. Assuming 15% of the cardiac output perfuses the head (with 12% in CoW), 5% supplies each arm and 75% supplies to the rest of the body through the thoracic aorta, the mean inlet flow rate of the heart is determined as:

$$Q_{h,m}=\frac{Q_{CoW,m}}{0.12}.$$ (5)

Consequently, the peripheral resistance of the rest terminal branches can be determined by the ratio of the pressure drop and the flow rate. For example, the peripheral resistance of thoracic aorta is calculated by:

$$R_{body,m}=\frac{\Delta P}{0.75Q_{h,m}},$$ (6)

To minimize the reflection at the terminal branch, *R*1 in WK3 is set as the characteristic impedance of the terminal branch,

$$R1=\frac{\rho c_{0}}{A_{0}}$$ (7)

with *c*_0_ is the speed of pulse wave propagation; *A*_0_ is the cross-section area of the segment. Then *R*2 is calculated as:

$$R2=R_{p}-R1.$$ (8)

By using Eq. 4, we observed that for some cases, *R*_*p*_ may be smaller than *R*1, this may be caused by the uncertainty of TCD and CTA measurement, as well as the possible unexpected reflection due to the cerebral vasospasm. To avoid a negative resistance, we altered the resistance for those cases with:

$$R1=0.8R_{p}\;and\;R2=0.2R_{p}.$$ (9)

With optimal peripheral resistance and proper flow rate at the heart, the obtained mean flow rate and mean blood pressure matches the measurement very well.

### 2.4 Compliance Correction

In a cerebral arterial network system, the mean flux distribution is mainly governed by the peripheral resistance, whereas the oscillation pattern is dominated by wall compliance and peripheral compliance. The wall compliance can be adjusted by varying Young’s modulus of the segment. It is more difficult to determine the peripheral compliance because of the complex wave interaction. In the present study, the objective of the compliance correction is to match the systolic and diastolic blood pressures of the brachial artery and the systolic and diastolic cerebral blood flow velocity. Since Alastruey et al. (2007)’s model is employed as the reference model, the pressure pulse calibration can be achieved by scaling the reference model. Given a volumetric flux of the heart in the model, the amplitude of the pressure pulse is inversely proportional to the peripheral compliance. First, we computed the ratio of mean blood pressure and flow rate between patient and reference model with:

$$\alpha_{1}=\frac{\Delta PQ_{ref,m}}{\Delta P_{ref}Q_{h,m}};$$ (10)

where Δ*P* is the pressure difference between *P*_*s*_ and *P*_*d*_, *Q*_*ref*,*m*_ and Δ*P*_*ref*_ are the reference mean flow rate and pressure difference by the reference model, and *Q*_*ref*,*m*_ = 92.6 ml s^−1^ and Δ*P*_*ref*_ = 55 mmHg that is obtained from the Simulink model. The Young’s modulus and the peripheral compliance of the segments can be altered by equation:

$$E_{1}=\alpha_{1}E_{ref}\;and\;C_{p,1}=\alpha_{1}C_{p,ref}$$ (11)

where *E*_*ref*_ and *C*_*p*,*ref*_ represent the Young’s modulus and the peripheral compliance in the reference model. With the first calibrated peripheral compliance and Young’s modulus, the tentative simulation of the Simulink model is implemented. This simulation can result in a good approximation of the pulse in the aorta arteries, but may not derive a satisfactory waveform in the cerebral arteries, i.e., poor agreement with *V*_*s*_ and *V*_*d*_ in CoW, which requires a second calibration. In intra-aortic hemodynamics simulation, multiple TCD measurement can be approached by iteratively adjusting the peripheral compliance with WK3, as demonstrated by Pant et al. (2014), Bonfanti et al. (2019), while wall compliance is kept constant during the iteration. However, the intracranial arteries are stiffer than extracranial arteries, the waveform in the ICA and BA are therefor not sensitive to the peripheral compliance of the terminal branch of the CoW. Hence, the second calibration is carried out by only adjusting the Young’s modulus of the cerebral arteries by an uniform factor *α*_2_, which is estimated by:

$$\alpha_{2}=\frac{\Delta Q_{ICAL,m}+\Delta Q_{ICAR,m}+\Delta Q_{BA,m}}{\Delta Q_{ICAL,n}+\Delta Q_{ICAR,n}+\Delta Q_{BA,n}},$$ (12)

where Δ*Q* is the flow rate difference between *Q*_*s*_ and *Q*_*d*_, with subscripts *m* and *n* refers to the measurement and numerical results respectively. The Young’s modulus and the peripheral compliance of the cerebral arterial segments can be altered by equation:

$$E_{2}=\alpha_{2}E_{1}\;and\;C_{p,2}=\alpha_{2}C_{p,1}.$$ (13)

Although individual calibration per arterial segment can be done with Kalman filter Pant et al. (2014), it required many iterations that made the computation inefficient. We applied an uniform factor because the flow velocity results by the tentative simulation in all cerebral segments was observed having similar trend comparing to the TCD measurement. Satisfactory results can be obtained without any complicated iteration.

### 2.5 Model Validation Metrics

Model validation is quantified with a number of statistical measures. The intraclass correlation coefficient (ICC) was used to assess the agreement between the results obtained by numerical simulation and the TCD measurement. Following the instruction by McGraw and Wong (1996), we selected the type of ICC(A,1) in the study, which assessed the degree of absolute agreement among simulation and measurement. The Taylor diagram (Taylor, 2001) is used to evaluate the validity of the proposed calibrations by comparing the numerical results to that by conventional boundary condition. Taylor diagram is a mathematical diagram to quantify the degree of correspondence between the modelled and observed behavior with three statistics: the Pearson correlation coefficient *ρ*, the centred-root-mean-square error *E*′, and the standard deviation *σ*, which was intensively used in climate study (Kärnä and Baptista, 2016). Based on the definition of the three statistics, they have the following relation:

$$E^{\prime 2}=\sigma_{m}^{2}+\sigma_{n}^{2}-2\sigma_{m}\sigma_{n}\rho .$$ (14)

where *n* demotes the variable by the numerical model; *m* demotes the measured counterpart. Making use of the law of cosines, Eq. 14 can be interpreted as geometrical relationship of a triangle, with *E*′, *σ*_*m*_, and *σ*_*n*_ are the length of the sides of the triangle, and *ρ* is the angle between sides *σ*_*m*_, and *σ*_*n*_. In order to compare the data sets at multiple arterial segments in the one diagram, the normalized Taylor diagram was employed by scaling Eq. 14 with *σ*_*m*_. Thus, the measurement perfectly locates at $\left(R=1,\theta =0\right)$ . The numerical results are displayed in polar coordinate with radial coordinate *r* = *σ*_*n*_/*σ*_*m*_ and angle *θ* = arccos *ρ*.

## 3 Results

### 3.1 Validation of Simulink Model

**FIGURE 4:**
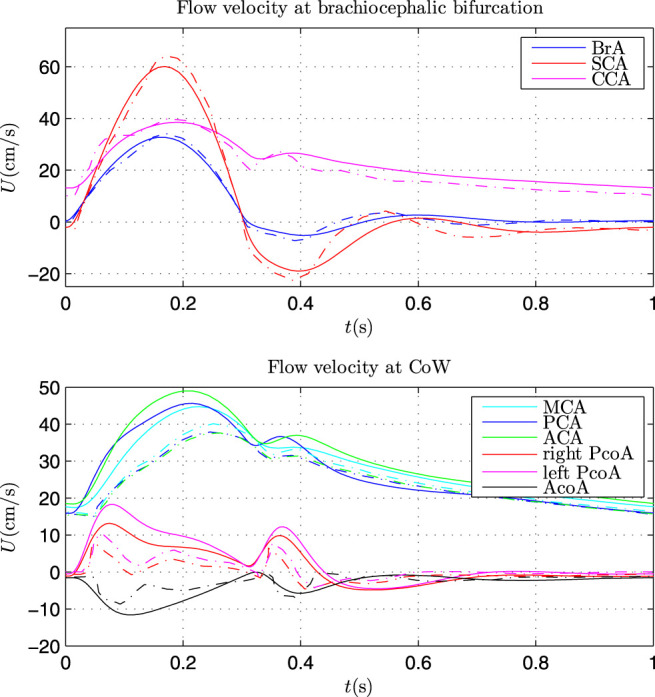
Comparison of flow velocity at brachiocephalic bifurcation (top) and in CoW (bottom) by Simulink model (solid lines) and 1D model (dash lines).

The Simulink model was firstly validated by comparing the results with that of 1D model by Alastruey et al. (2007), as presented in Figure 4.

The same parameters as that in Alastruey et al. (2007) were applied in the Simulink model. The flow velocity at the brachiocephalic (BrA), right subclavian (SCA) and right common carotid (CCA) showed very good agreement with that by 1D model. Some discrepancy can be found for the flow velocity in CoW. There may be two main causes: 1) wave propagation in the CoW was complex and the waveform was vulnerable to wave interference; 2) the Simulink model is a lumped model, which can be regarded as the first order discretisations of 1D systems (Milišić and Quarteroni, 2004), omitting the (nonlinear) convective acceleration term. Nevertheless, the Simulink model can capture the main waveform similar to that by 1D model, and the waveform can be further calibrated with the TCD measurement. Hence, the Simulink model can be used to investigate the patient-specific cerebral hemodynamics.

### 3.2 Patient-specific Simulation

**FIGURE 5:**
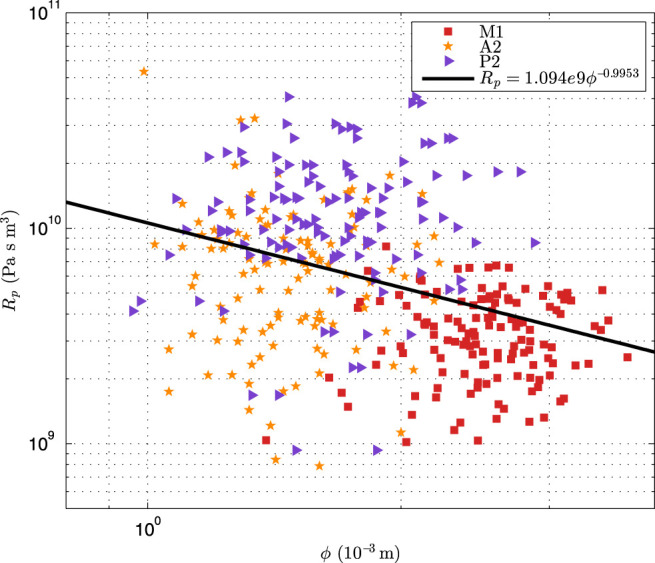
The log-log scatter plot of the optimized peripheral resistance against the diameter of the afferent segments of CoW.

In this study, 66 sets of patient-specific simulation were implemented. Figure 5 presents the log-log scatter plot of the optimized peripheral resistance obtained by Eq. 4 against the diameter of afferent segments of CoW, with the black line showing the log-log regression.

**FIGURE 6:**
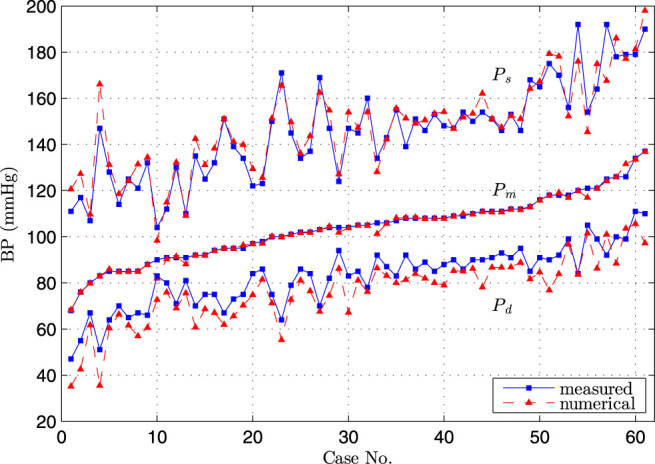
Comparison of diastolic, systolic and mean blood pressure at brachial artery by simulation and measurement, the cases were sorted in order of low to high measured mean blood pressure.

It can be seen that the optimized peripheral resistance showed a trench of inverse-proportional to the diameters (*R*_*p*_ ∝ *ϕ* ^−0.995 3^), but it was highly scattering. Alastruey et al. (2007) assumed that the peripheral resistance was inverse-proportional to the cross-sectional areas (*R*_*p*_ ∝ *ϕ* ^−2^). This suggested that this simple assumption was not valid for the patient-specific simulation as the peripheral resistance dominated the flow distribution in the CoW. In addition, the resistance of A2 segment was larger than that of P2 with similar diameter, implying that the resistance in the anterior cerebral circulation was larger than that in the posterior cerebral circulation, that was not surprise as the blood velocity in ICA was commonly larger than that in BA. Figure 6 showed the comparison of blood pressure after the first compliance correction, i.e., Eq. 13.

The excellent agreement was obtained between the numerical and measured *P*_*m*_, and the very good agreement was observed for *P*_*s*_ and *P*_*d*_. Since these three parameters were commonly used to sketch the blood pressure waveform, we considered that the obtained pressure waveform by the Simulink model at the entrance of CoW represented the hemodynamic condition at the time of TCD measurement.

**FIGURE 7:**
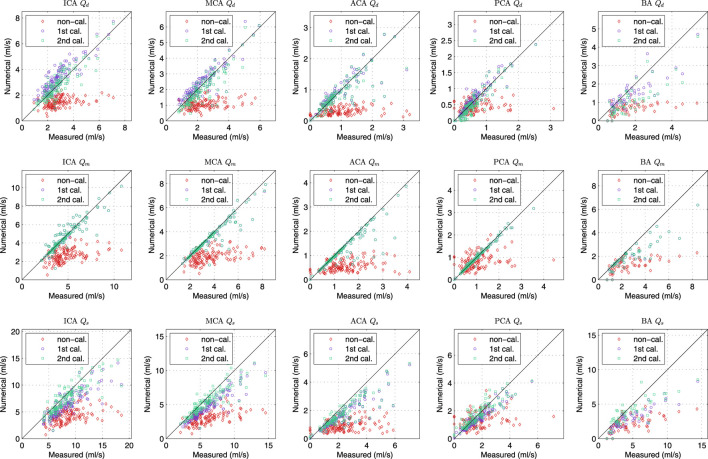
Comparison of diastolic (1st row), mean (2nd row) and systolic (3rd row) flow rate of various cerebral segments by measurement and various simulations.

Figure 7 showed the comparison of flow rate in the afferent and efferent arterial segments between the measurement and the results by three simulations. The measured flow rate is indirectly obtained by solving the optimization problem of peripheral resistance, i.e., Eq. 4. In the “non-calibrated” simulation, we calculated the peripheral resistance and compliance in CoW of each patient based on the assumption that the blood flux of each part in the body followed a given flow distribution, and the outflow of the CoW was proportional to their initial cross-sectional areas. The agreement between the measurement and the “non-calibrated” simulation was poor, indicating that the simple assumption was not valid for the patient-specific study. The customized parameter must be applied per patient to represent the patient’s hemodynamics at the time of measurement. In the “first calibrated” simulation, the calibrated peripheral resistance and compliance in the body segments was applied in the simulation. The mean flow rate in CoW by simulation agreed well with the measurement, and the results of the efferent segments outperformed that of the afferent segments. The simulation slightly underestimated the diastolic flow rate while overestimated the systolic flow rate, implying that the amplitude of the waveform was lower in simulation. In the “second calibrated” simulation, the compliance of the cerebrate segments were calibrated based on the first calibrated results. The calibration did not alter the mean flow rate in the previous simulation, while the agreement of the diastolic and systolic flow rate was improved. Since TCD-measurement has been used to calibrate the model as well as to validate the model output, it is not surprising that the flow rate by the “calibrated” simulation yields the best agreement. Based on the calibrated model, we can reproduce the cerebral hemodynamic condition for the patient at the time of measurement and provide more comprehensive cerebral hemodynamics for the clinician.

The statistic of the results in Figure 7 were summarized in Table 1. The agreement of the second calibrated results was good (0.75 < *ICC* < 0.90) to excellent (*ICC* > 0.90), except the BA. The current arterial network did not take into account the minor branches on the CoW that would introduce more error on the BA than that other segments as the BA is a relatively short segment but has the most minor branches. *Q*_*m*_ always presented better agreement comparing to *Q*_*d*_ and *Q*_*s*_, this was because *Q*_*m*_ was strongly dominated by the peripheral resistance (the peripheral resistance was generally two order larger than the segmental resistance of the CoW). *Q*_*d*_ and *Q*_*s*_ was not only influenced by the peripheral compliance but also the compliance of the segment in CoW. Nevertheless, the agreement was satisfactory, particularly considering that only twice calibration were applied, which was significantly less iteration than using EnKF.

**FIGURE 8:**
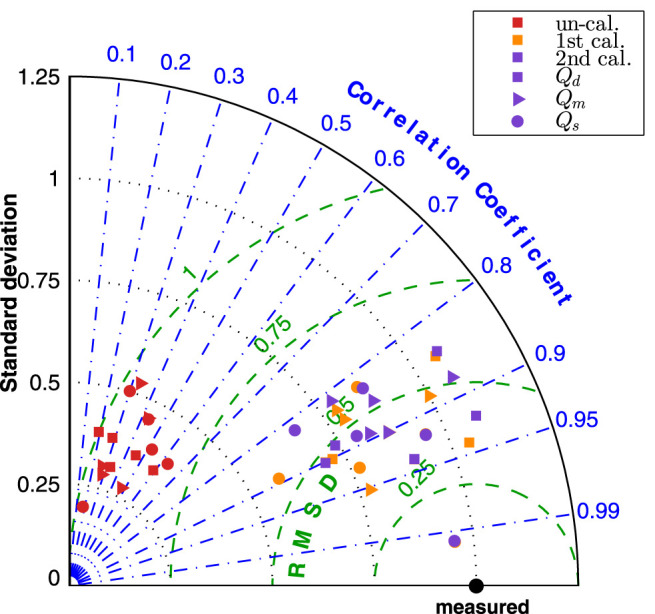
Normalized Taylor diagram of various celebrate segments by three simulations.

Figure 8 presented the normalized Taylor diagram of various segments that gave an impression of importance of the calibration procedures. The results by the three simulations were plotted in different colour, and three marker symbols were used to distinguish the results of *Q*_*d*_, *Q*_*m*_ and *Q*_*d*_. In the Taylor diagram, good results were indicated by having relatively high correlation coefficient and low RMSE (as close to the measured point as possible). The agreement of the “un-calibrated” simulation was poor as it has very low correlation coefficient and very high RMSE, while the first and second calibrated results are much closer to the measurement.

**FIGURE 9:**
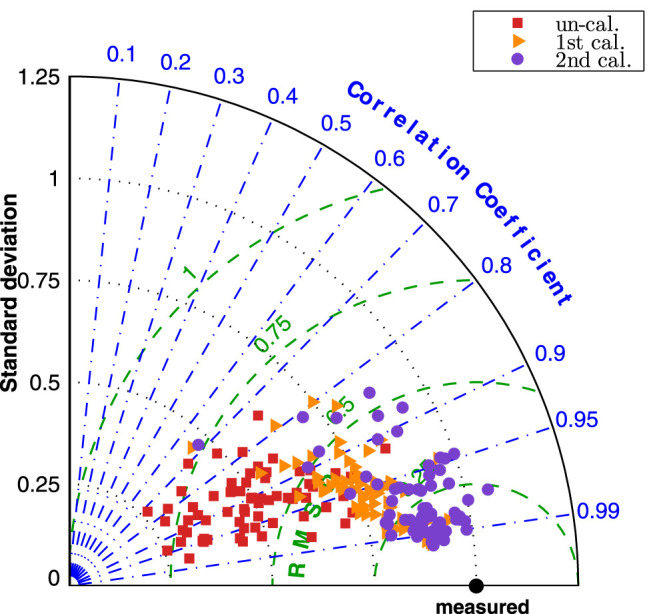
Normalized Taylor diagram of each patient by three simulations.

The improvement by the calibration procedures can be seen from Figure 9, the normalized Taylor diagram of flow rate at 9 cerebral locations per patient. The data points cluster of the second calibrated results located at around the ∼0.25 RMSE circle, while the first calibrated results shifted further from the measurement, indicating that the second calibration can describe the waveform pattern better than the first calibration.

## 4 Discussion

When mentioning patient-specific cerebral simulation, many studies refer to the patient-specific geometry of arteries but do not take patient-specific boundary conditions into account. Our results have demonstrated the importance of boundary conditions and wall properties. Comparison by three simulations confirmed the findings of Hillen et al. (1988) that the peripheral resistance plays a predominant role in the flow distribution of the efferent arteries of the CoW. The blood flow waveform in segments in the CoW is not significantly influenced by the peripheral compliance, but would be adapted to any change of the segmental elasticity in the cerebral arterial network. To reproduce the hemodynamic environment in cerebral vasospasm, including the pulse waveform, patient-specific boundary conditions and wall properties must be corrected by integrating flow measurement. On the other hand, the model calibration relies on flow measurement on cerebral segments, which may be missing in retrospective studies, sometimes difficult to be obtained for some patients. The uncalibrated model cannot derive a reliable absolute blood flow rate, as shown in Figure 8 that the correlation coefficient is too low. Therefore, the absolute quantitative comparison should be avoided in the statistical analysis. Alternatively, using the flow rate ratio between the segments may alleviates some of this problem and yield more meaningful results, as it can be seen from Figure 9 that although the universal boundary condition leads to the worst results, there is not any significant difference of the correlation coefficient by three simulations. This idea is similar to the study by Lindegaard et al. (1988) that used the ratio of blood velocity in MCA and ICA by TCD to diagnose vasospasm.

The present model with personalized calibration has potential clinical applications. The model integrates the patient’s morphology information and available flow measurement to provide the comprehensive cerebral hemodynamics, which may help the clinician to establish the correlations between hemodynamic indices and cerebrovascular disease. Since cerebral vasospasm due to the aSAH can result in delayed cerebral ischemia, the model can provide insight in cerebral hemodynamic development during the course of vasospasm. It may also be easily employed to predict the improvement of the blood supply after intracranial bypass operation or treatment of the carotid artery stenosis, thanks to the intuitive Simulink model. In addition, the model can also provide the boundary conditions for a patient-specific CFD model to investigate the detailed local flow in the CoW, such as the flow pattern in intracranial aneurysms. We consider the present work as the first step toward a numerical computational framework serves as a clinical decision-making tool for hemodynamic related cerebrovascular disease. The major advantage of the present model is that the model was developed with a block diagram, which is also user-friendly to the clinician. Also, the required patient’s data are provided by CTA and TCD, which is commonly accessible clinical data. As such, it is easy to build up a patient-specific simulation. The calibration procedure is implemented to correct the model input with a moderate computational burden.

The present study also has limitations. The most significant limiting factor of the model is the use of indirectly obtained flow rate for calibration and validation. TCD measures flow velocity at unknown location of the target segment, the flow rate is numerically determined with a linear model of the CoW network. On one hand, the linear model obeys the law of conservation of mass and integrates the TCD velocity and dimension of segments by CTA, which estimates the flow distribution in the CoW network with minimal mass conservation error. On the other hand, the linear model uses simplified CoW network omitting the less important branch, which introduces uncertainty into the model. Finally, while we have identified the importance of the calibration procedure for patient-specific simulation, additional patient information (arterial blood pressure, TCD records) is required for calibration, which may limit its application. Nevertheless, we assume that the required data is commonly available for patients with cerebrovascular disease.

## 5 Conclusion

This paper presents a three-steps approach for implementation of a patient-specific cerebral blood flow simulation based on commonly available clinical datasets. The new approach integrates TCD and CTA data to correct the input parameters of an object-oriented hemodynamic model. Simulation with 66 sets of patient data was carried out to evaluate the validity of the model. The results demonstrated the importance of the peripheral resistance in the patient-specific cerebral blood flow simulation, and the satisfactory pulse waveforms can be reconstructed by the personalized model calibration.

The proposed method can reproduce the blood flow in the CoW where the blood flow in afferent and efferent segments was in good agreement with the TCD measurement. The model is potentially a promising tool for developing clinical understanding of cerebrovascular disease. The obtained results will be further investigated by clinical researcher to find the potential relationship between the vasospasm and the configuration of the CoW.

## Data Availability

The raw data supporting the conclusion of this article will be made available by the authors, without undue reservation.
